# Geographic variation in supply, demand, and adequacy of the obstetrics and gynecology physician workforce: forecasts and shortage risks in the United States

**DOI:** 10.1007/s00404-026-08322-5

**Published:** 2026-02-14

**Authors:** Jason Silvestre, Gweneth B. Lazenby

**Affiliations:** Medical University of South Carolina, 96 Ashley Ave, Charleston, SC 29425 USA

**Keywords:** Obstetrics, Gynecology, Workforce, Shortage, Inadequacy

## Abstract

**Purpose:**

This study assessed geographic variations in the supply, demand, and adequacy of the United States (US) obstetrics and gynecology physician (OGP) workforce.

**Methods:**

This was a cross-sectional analysis of OGPs using the Health Workforce Simulation Model. Supply and demand were defined as the numbers of full-time equivalent (FTE) OGPs working and needed, respectively. Adequacy was defined as the ratio of supply to demand. Comparisons were made using Chi-squared tests, and linear regression was used to analyze OGP workforce trends.

**Results:**

From 2025 to 2037, the demand for OGPs is projected to increase (52,620–54,020 FTEs, 2.7% increase, *p* < 0.001) while the supply of OGPs is projected to decrease (49,170–44,130 FTEs, 10.3% decrease, *p* < 0.001). As a result, OGP workforce adequacy is projected to decrease over the study period from 93.4% to 81.7% (*P* < 0.001). By 2037, the West had the lowest OGP workforce adequacy and the Northeast had the highest adequacy (74.4% vs 98.6%, *P* < 0.001). Non-metropolitan areas were projected to have lower OGP workforce adequacy than metropolitan areas (51.4% vs 85.1%, *p* < 0.001). The states with the lowest projected OGP workforce adequacy were Utah (49.3%), Idaho (51.5%), and Arizona (58.3%) in 2037.

**Conclusion:**

OGP workforce supply is expected to fall short of anticipated demand, with uneven geographic distribution across the US. Addressing this imbalance will require strategic planning to expand the OGP workforce equitably, especially in non-metropolitan areas, the West, and certain identified states like Utah and Idaho.

## What does this study add to the clinical work

**Table:** 

Geographic disparities exist in the distribution of obstetrics and gynecology physicians across the US with notable deficiencies in non-metropolitan areas and the West. Future state-level efforts are needed to augment the supply of obstetrics and gynecology physicians in areas with identified deficiencies.	

## Introduction

The obstetrics and gynecology physician (OGP) workforce in the United States (US) is experiencing increasing pressures amidst rising patient demand, an aging physician population, and evolving practice patterns [1–3]. Recent epidemiologic and demographic trends, including population growth among reproductive-age individuals and increasing complexity of maternal health needs, have intensified the clinical workload in both obstetric and gynecologic care [4–6]. At the same time, a significant proportion of the current OGP workforce is approaching retirement age, and the training capacity of graduate medical education (GME) residency programs in obstetrics and gynecology has not expanded to meet anticipated needs [7, 8].

Previous studies have signaled a potential mismatch between the supply of practicing OGPs and the demand for their services, particularly in rural and underserved regions [9–11]. However, there remains a paucity of contemporary research using national, validated datasets to understand the projected supply, demand, and adequacy of OGPs across the US. Without a comprehensive understanding of future OGP workforce dynamics, policy makers, educators, and health systems may be inadequately equipped to address increasing access issues experienced in women’s healthcare.

The Health Resources and Services Administration (HRSA) is a federal agency charged with ensuring access to healthcare services for medically underserved and vulnerable patient populations across the US [12]. In 2016, the HRSA created the Health Workforce Simulation Model (HWSM) as a forecasting tool to estimate national healthcare workforce supply and demand scenarios [13]. To date, a comprehensive analysis of OGPs using the HWSM is void in the literature. Thus, the purpose of this study was to analyze US federal government projections regarding the supply, demand, and adequacy of OGPs in the US. Based on prior literature [4–6, 9–11], we hypothesized that OGP workforce adequacy would decrease over time. Additionally, we hypothesized that OGP workforce adequacy would be lowest in non-metropolitan areas and certain identifiable states.

## Materials and methods

This was a cross-sectional study of US-based OGPs utilizing data from the HWSM. Data and assumptions in the HWSM were derived from multiple data sources including the US Census [14], American Medical Association (AMA) [15], Centers for Medicare and Medicaid Services (CMS) [16], and Healthcare Cost and Utilization Project (HCUP) [17]. Methodologies employed by the HWSM to project physician workforce adequacy have been described in depth previously and summarized briefly below [18]. Definitions for geographies including regions (Northeast, West, Midwest, South) and metropolitan designations (metropolitan, non-metropolitan) adhered to those of the US Census [14]. This study qualified for review exemption based on the policies of the institutional review board. This study also adhered to the observational reporting standards of the Strengthening the Reporting of Observational Studies in Epidemiology [19].

The HWSM utilizes a dynamic stock-and-flow microsimulation framework to model the supply and demand for OGPs in the US. The HWSM operates by simulating the behavior of one individual, either a patient or a physician within a larger, statistically representative cohort of patients and physicians across the US. In this study, supply was defined as the number of full-time equivalent (FTE) OGPs working in the US. Supply projections began with a baseline estimate of the current OGP workforce utilizing data from the AMA Physician Masterfile and state medical licensing board data [15, 16]. Data from the American Association of Medical Colleges (AAMC) National Sample Survey of Physicians indicated that most OGPs work greater than forty hours per week [20]. Accordingly, supply predictions in the HWSM are intentionally greater than the count of active OGPs to account for these differences. To model annual entrants for supply, certification and licensing data were integrated from the Accreditation Council for Graduate Medical Education (ACGME) [21]. Annual entrants into the OGP workforce were projected based on residency training capacity during the 2022–2023 academic year [21]. Assumptions for career duration and attrition among OGPs were obtained from the AAMC National Sample Survey of Physicians [20]. State-level licensing records were utilized to model attrition due to retirement, mortality, and career changes [15]. Patterns of OGP migration were sourced from the CMS National Plan and Provider Enumeration System to model workforce attrition [16]. The synthesis of data across multiple sources allows the HWSM to build reasonable estimates for the future supply of OGPs.

Demand was defined as the number of FTE OGPs required to maintain current levels of healthcare service utilization. Demand estimates for OGPs were derived from the Medical Expenditure Panel Survey, the National Ambulatory Medical Care Survey, and other HCUP datasets that link healthcare visits with patient demographics, clinical characteristics, and insurance status [17]. In the status quo demand scenario, the HWSM assumes that current healthcare utilization patterns by age, sex, insurance type, and metropolitan status are preserved into the future. Demand for OGPs is stratified by site of care delivery, including the office, outpatient settings, inpatient settings, and the emergency room. Population growth estimates from the US Census [14] were used to model population growth for patients requiring OGP services, while physician staffing ratios were preserved from the American Medical Group Association [22]. The Medical Expenditure Panel Survey was used to generate healthcare prediction equations [23]. Healthcare administrative codes were further used to model growth in OGP demand, including ICD-9 diagnosis (614–679, V22–V24), ICD-9 procedure (72–75), and ICD-10 diagnosis (N70–N98, O00–O9A) codes [17].

Primary outcomes of interest were defined as supply, demand, and adequacy of the US OGP workforce, utilizing the methodology employed by a recent vascular surgery workforce study [24]. All projected years from the HWSM were extracted on July 1, 2025, which included the years 2025–2037. Linear regression modeling was used to analyze workforce trends over the study period based on similar statistical techniques employed in the HWSM. OGP workforce adequacy was defined as the ratio of supply to demand multiplied by 100%. Percentage differences over the study period were calculated for primary outcomes. Chi-squared tests were used to make geographical comparisons of OGP workforce adequacy. All statistical tests were two-tailed and calculated using GraphPad Prism© software (San Diego, CA). P values less than 0.05 were considered statistically significant.

## Results

### Trends in the supply, demand, and adequacy of the obstetrics and gynecology physician workforce

**Fig. 1:**
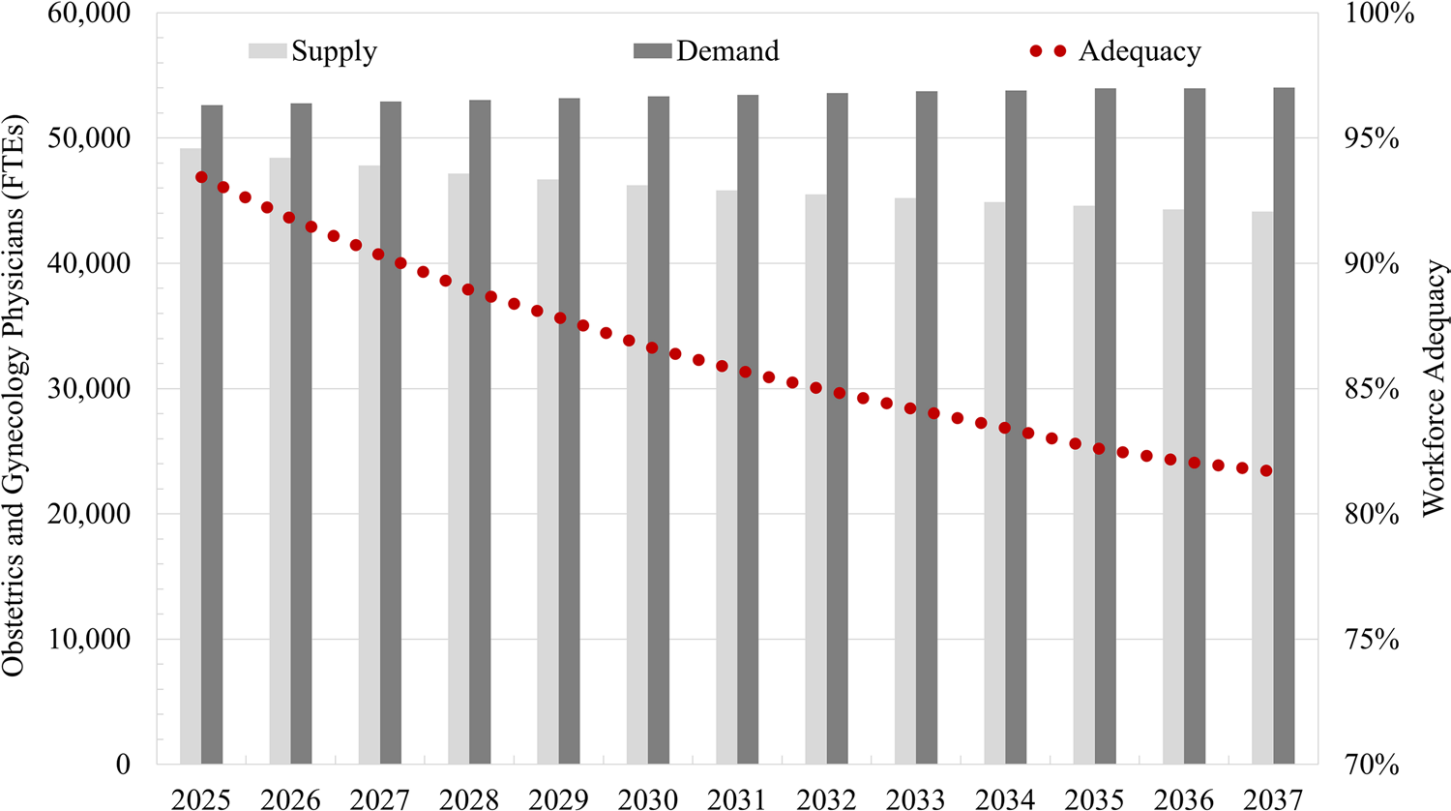
Supply, demand, and adequacy of the obstetrics and gynecology physician workforce in the United States. A bar graph demonstrating the supply and demand of obstetrics and gynecology physicians (OGPs) on the left, and a line graph demonstrating OGP workforce adequacy on the right

In 2025, the national supply of OGPs was 49,170 FTEs, and it was projected to decrease to 44,130 FTEs by 2037 (10.3% decrease, *P* < 0.001, Fig. 1). Over the same period, the national demand for OGPs was projected to increase from 52,620 to 54,020 FTEs (2.7% increase, *P* < 0.001). Consequently, OGP workforce adequacy was projected to decrease over the study period from 93.4% to 81.7% (11.7% decrease, *P* < 0.001).

### Comparison of obstetrics and gynecology physician workforce adequacy by US region

**Fig. 2:**
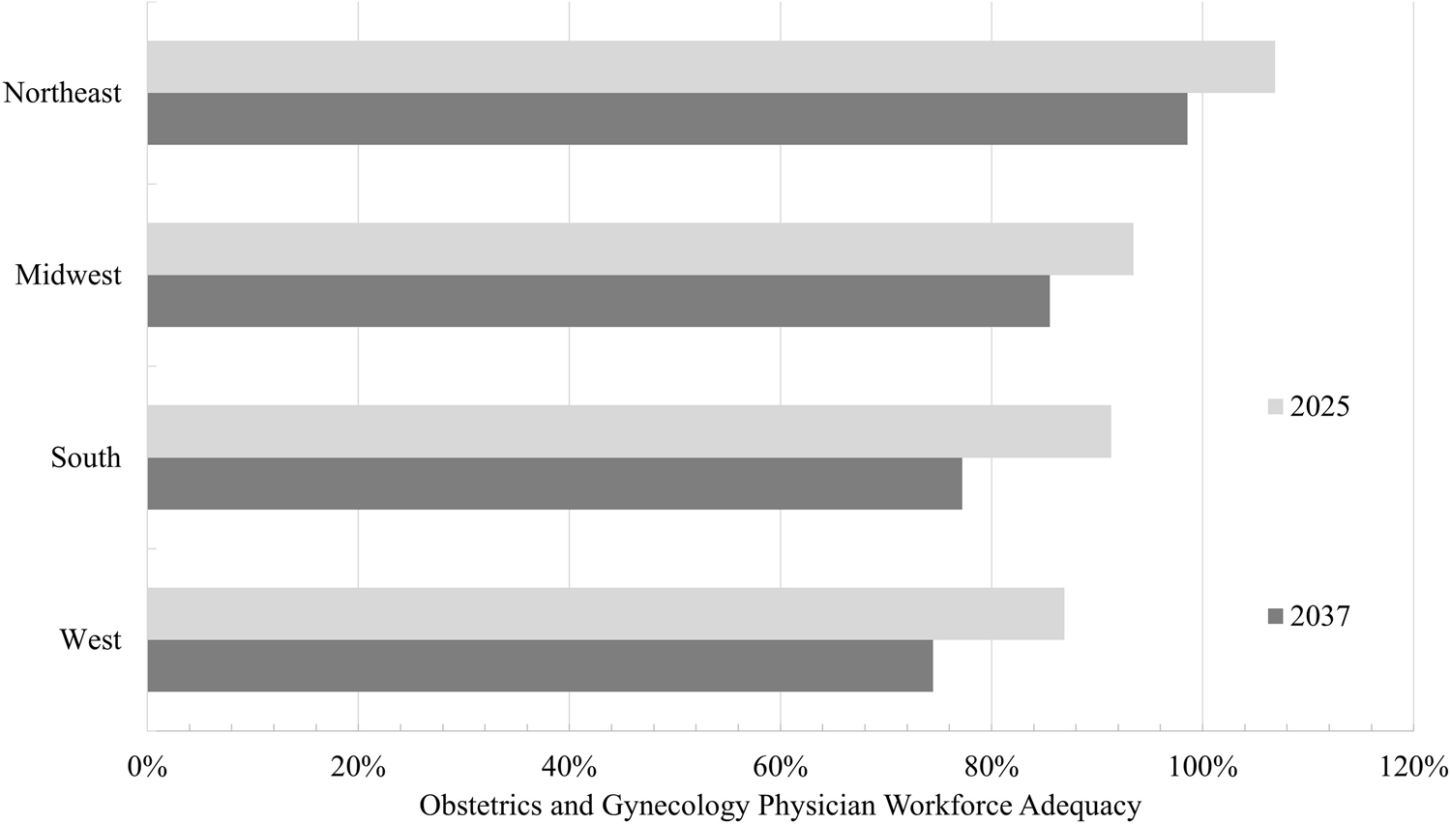
Obstetrics and gynecology physician workforce adequacy by United States Region. Double bar graph demonstrating obstetrics and gynecology physician (OGP) workforce adequacy from 2025 to 2037; Chi-squared tests demonstrate significant decreases in OGP workforce adequacy across all regions

Geographic disparities existed in OGP workforce adequacy over the study period (Fig. 2). In 2025, OGP workforce adequacy was the lowest in the West and the highest in the Northeast (86.9% vs 107%, *P* < 0.001). By 2037, OGP workforce adequacy was projected to remain the lowest in the West and be the highest in the Northeast (74.4% vs 98.6%, *P* < 0.001). Across all US regions, OGP workforce adequacy was projected to decrease over the study period (*P* < 0.001), including the South (14.1% decrease), the West (12.5% decrease), the Northeast (8.3% decrease), and the Midwest (7.9% decrease).

*Comparison of* the *obstetrics and gynecology physician workforce adequacy by US metropolitan status.*

**Fig. 3:**
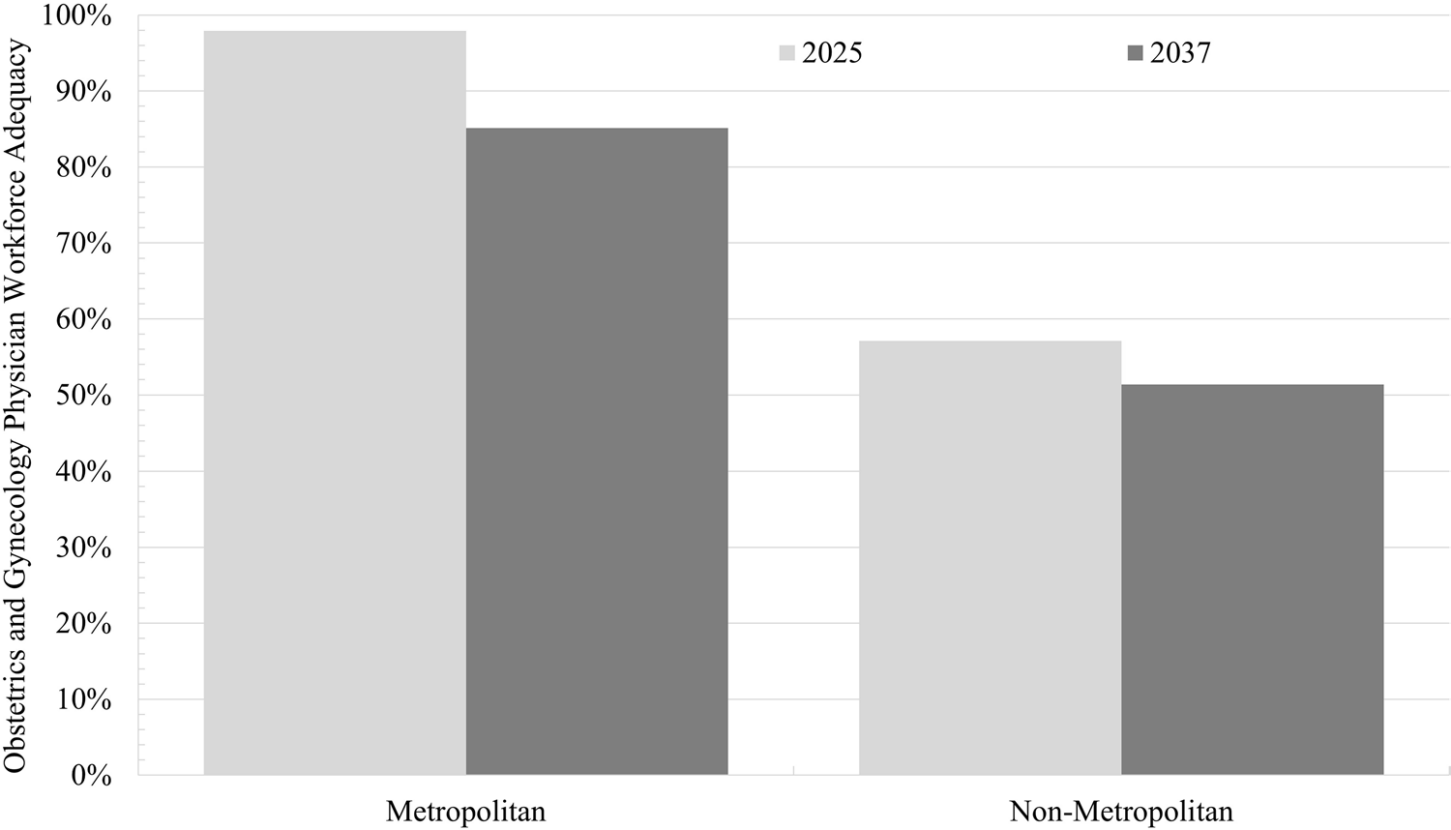
Comparison of obstetrics and gynecology physician workforce adequacy in metropolitan and non-metropolitan areas. Double bar graph demonstrating obstetrics and gynecology physician (OGP) workforce adequacy from 2025 to 2037; Chi-squared tests demonstrate significant decreases in OGP workforce adequacy in both metropolitan and non-metropolitan areas

In 2025, OGP workforce adequacy was lower in non-metropolitan areas than in metropolitan areas (57.1% vs 97.9%, *P* < 0.001) (Fig. 3). By 2037, OGP workforce adequacy was projected to remain lower in non-metropolitan areas than in metropolitan areas (51.4% vs 85.1%, *P* < 0.001). OGP workforce adequacy was projected to decrease in both metropolitan areas (12.8% decrease) and non-metropolitan areas (5.8% decrease) over the study period (*P* < 0.001).

### Disparities in obstetrics and gynecology physician workforce adequacy by State

**Fig. 4:**
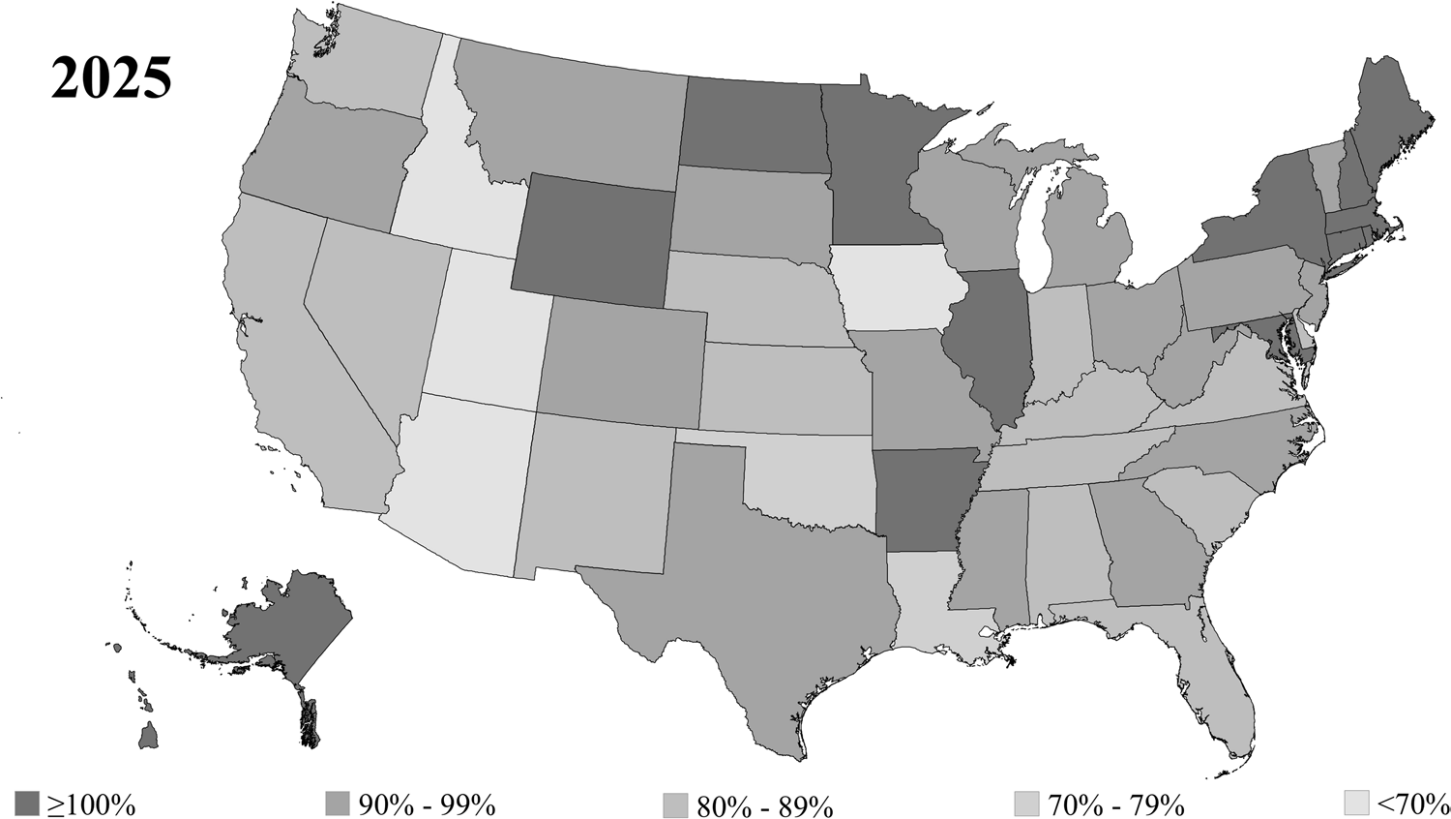
State-level disparities in obstetrics and gynecology physician workforce adequacy in 2025. Geographic heat-map demonstrating obstetrics and gynecology physician (OGP) workforce adequacy in 2025

**Fig. 5:**
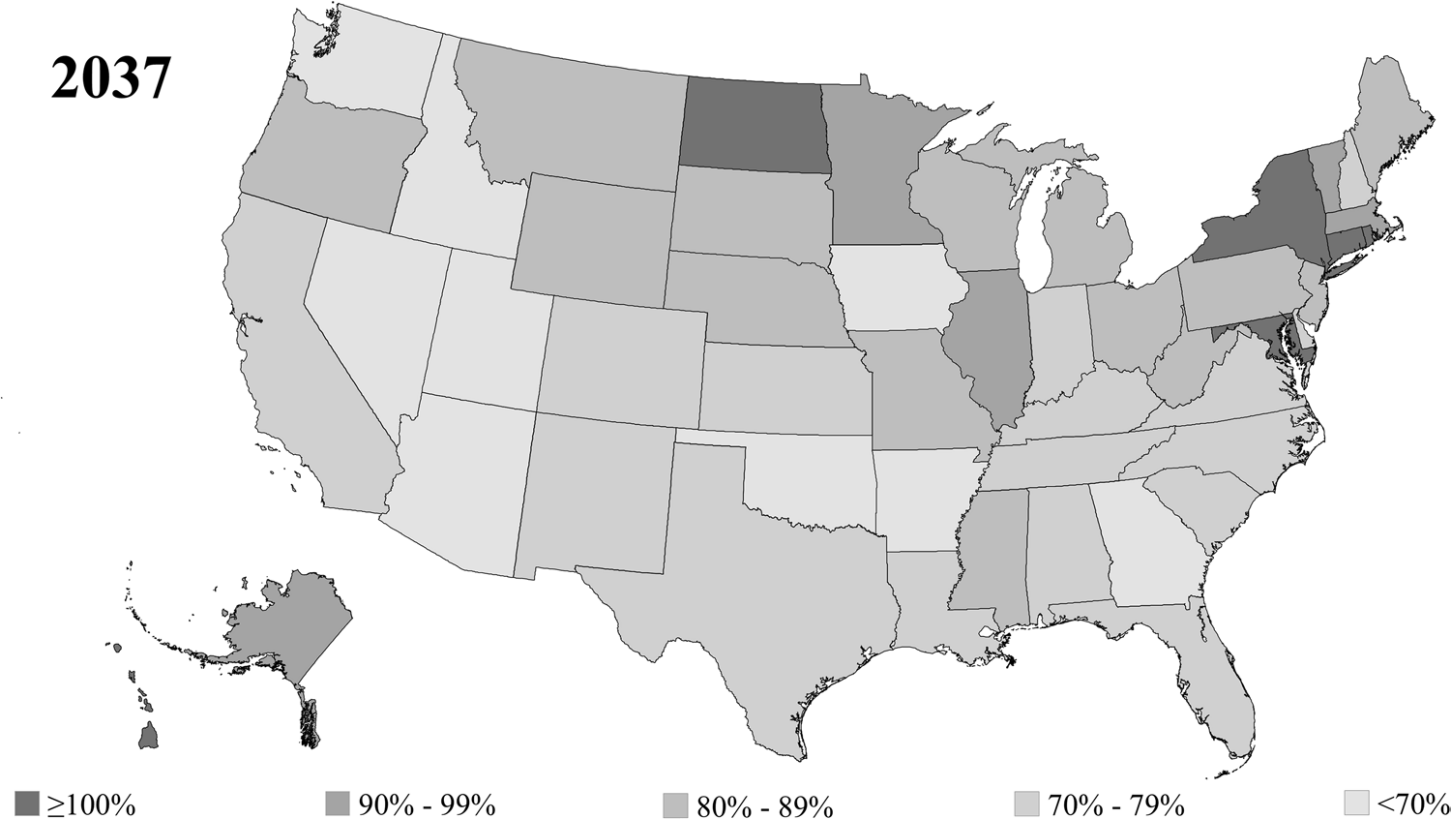
State-level disparities in obstetrics and gynecology physician workforce adequacy in 2037. Geographic heat-map demonstrating obstetrics and gynecology physician (OGP) workforce adequacy in 2037

In 2025, there were 36 states where OGP workforce adequacy was less than 100%, including three states where adequacy was less than 70% (Fig. 4). These states included Utah (65.6%), Idaho (66.7%), and Iowa (68.8%). By 2037, there were 42 states where OGP workforce adequacy was projected to be less than 100%, including nine states where adequacy was less than 70% (Fig. 5). These states included Utah (49.3%), Idaho (51.5%), Arizona (58.3%), Iowa (61.7%), Arkansas (63.8%), Nevada (64.0%), Oklahoma (65.6%), Washington (68.6%), and Georgia (69.4%).

In 2025, the states and districts with the highest OGP workforce adequacy were the District of Columbia (193%), Hawaii (135%), New York (118%), and Connecticut (118%). By 2037, the states and districts with the highest projected OGP workforce adequacy were the District of Columbia (180%), New York (116%), Hawaii (116%), and Vermont (111%) (Table 1).

**Table 1:** Trends in the supply, demand, and adequacy of the obstetrics and gynecology physician workforce. List of the supply and demand of obstetrics and gynecology physicians (OGPs) in full-time equivalents (FTEs) across states, which are ranked from least to most adequate in 2037

State or district	2025			2037		
	Supply	Demand	Adequacy	Supply	Demand	Adequacy
Utah	400	610	65.6%	360	730	49.3%
Idaho	200	300	66.7%	170	330	51.5%
Arizona	880	1,170	75.2%	770	1,320	58.3%
Iowa	330	480	68.8%	290	470	61.7%
Arkansas	330	460	71.7%	300	470	63.8%
Nevada	380	470	80.9%	320	500	64.0%
Oklahoma	430	610	70.5%	400	610	65.6%
Washington	1,060	1,310	80.9%	940	1,370	68.6%
Georgia	1,590	1,720	92.4%	1,340	1,930	69.4%
Florida	2,900	3,350	86.6%	2,550	3,610	70.6%
Alabama	620	730	84.9%	530	740	71.6%
Tennessee	980	1,100	89.1%	830	1,140	72.8%
Kentucky	570	700	81.4%	520	710	73.2%
Kansas	360	440	81.8%	330	450	73.3%
New Mexico	260	310	83.9%	230	310	74.2%
Virginia	1,280	1,430	89.5%	1,150	1,530	75.2%
Delaware	140	170	82.4%	130	170	76.5%
South Carolina	750	850	88.2%	690	900	76.7%
Texas	4,050	4,460	90.8%	3,840	5,000	76.8%
New Hampshire	220	230	95.7%	170	220	77.3%
Colorado	960	1,020	94.1%	890	1,130	78.8%
Indiana	960	1080	88.9%	850	1070	79.4%
North Carolina	1,660	1,720	96.5%	1,490	1,870	79.7%
California	5,630	6,310	89.2%	5,110	6,400	79.8%
Maine	200	200	100%	160	200	80.0%
Nebraska	260	310	83.9%	240	300	80.0%
Michigan	1,520	1,600	95.0%	1,310	1,620	80.9%
Mississippi	370	410	90.2%	320	390	82.1%
South Dakota	110	120	91.7%	100	120	83.3%
West Virginia	220	240	91.7%	200	240	83.3%
New Jersey	1,450	1,460	99.3%	1,200	1,420	84.5%
Wisconsin	820	900	91.1%	730	860	84.9%
Oregon	720	730	98.6%	650	760	85.5%
Wyoming	70	70	100%	60	70	85.7%
Missouri	890	940	94.7%	810	940	86.2%
Montana	150	160	93.8%	140	160	87.5%
Pennsylvania	2,010	2,090	96.2%	1,830	2,090	87.6%
Ohio	1,760	1,890	93.1%	1,600	1,820	87.9%
Alaska	120	110	109%	100	110	90.9%
Minnesota	920	900	102%	870	940	92.6%
Massachusetts	1,270	1,260	101%	1,140	1,210	94.2%
Illinois	1,960	1,930	102%	1,740	1,800	96.7%
North Dakota	110	110	100%	100	100	100%
Connecticut	730	620	118%	620	610	102%
Maryland	1,170	1,020	115%	1,060	1,040	102%
Rhode Island	210	190	111%	200	190	105%
Louisiana	760	700	109%	730	670	109%
Vermont	110	100	110%	100	90	111%
Hawaii	270	200	135%	220	190	116%
New York	3,780	3,190	118%	3,430	2,950	116%
District of Columbia	270	140	193%	270	150	180%

## Discussion

This study confirmed our hypothesis that OGP workforce adequacy is projected to decline significantly between now and 2037. This occurred primarily due to projected declines in the future supply of OGPs, although demand for OGPs was expected to increase. As a result, OGP workforce adequacy was projected to decrease significantly over the study period by 11.7%. Additionally, significant geographic disparities in OGP workforce adequacy were identified, including in the West and in non-metropolitan areas. Certain states demonstrated the lowest OGP workforce adequacy, including Utah, Idaho, Arizona, and Iowa. Overall, results from this study highlighted several geographic disparities in the access to OGPs, which may have implications for US policy makers, state governments, and key specialty stakeholders including the American College of Obstetricians and Gynecologists (ACOG) and American Gynecological & Obstetrical Society (AGOS). Multiple factors likely contributed to the observed trends, including the anticipated retirement of OGPs in the US and population growth among reproductive-age individuals with greater maternal health needs in certain areas [5–7]. Understanding the variables that affect OGP workforce adequacy remains an important area of ongoing research. Ultimately, this study highlights an impending deficiency and maldistribution of the OGP workforce across the US, which may require multiple coordinated efforts to address moving forward.

The results of this study add to the previous literature highlighting growing concerns about the adequacy of the US OGP workforce [1–3]. Multiple reports have underscored the significant mismatch between OGP supply and patient demand over the coming decade, driven by factors such as OGP retirement, population growth, and increased complexity of patient care [9–11]. ACOG has projected a national shortage of up to 9,000 OGPs by 2030 with rural and underserved areas particularly affected [25]. Geographic maldistribution of the OGP workforce remains a persistent challenge as recent graduates often prefer to practice in urban or suburban areas [26] leaving non-metropolitan areas with limited access to maternity and gynecologic care. Additionally, the aging of the current OGP workforce combined with a limited GME training capacity for OGPs, further compounds the concern about long-term sustainability [8, 9, 27]. Previous studies have also highlighted the high degree of physician burnout and medico-legal pressures experienced by the OGP workforce [28, 29], which may lead to early retirement and reduced clinical working hours [30, 31]. Collectively, these trends have underscored the need for policy interventions such as expansion of GME training capacity and improvement of OGP recruitment incentives in underserved areas. However, given the severity of OGP deficiencies expected in underserved areas, there may be several opportunities to implement additional creative strategies to augment OGP workforce adequacy.

Deficiencies in the US OGP workforce pose significant risks to vulnerable patient populations, particularly in non-metropolitan and underserved communities [9–11]. Restricted access to OGPs can delay prenatal care, which may be linked to higher maternal and infant morbidity and mortality rates [32]. Women living in underserved areas may need to travel longer distances for routine or emergency care, which may increase the likelihood of adverse pregnancy outcomes, including preterm births and other labor and delivery complications [33, 34]. Beyond obstetric care, gynecologic services, including cancer screenings, contraceptive counseling, and management of chronic conditions like endometriosis and pelvic floor disorders, may often be inaccessible, thus contributing to poor long-term reproductive health outcomes [35]. The absence of women’s healthcare services can exacerbate health disparities among low-income populations, racial and ethnic minority populations, and patients without reliable transportation [36]. Inadequate access to OGP services may contribute to widening health disparities and lead to increased healthcare costs and emergency services utilization due to preventable complications [36]. Thus, addressing US OGP workforce deficiencies is not only important for improving individual health outcomes, but remains critical to promoting public health standards and addressing widening gaps in health equity.

Based on this study, there are several recommendations that are worthy of additional research and consideration to address the decreasing supply of OGPs (Table 2). First, there may be a need to increase the GME training capacity for OGPs especially in underserved areas [8]. The expansion of rural-track or community-based training programs may encourage OGP retention in non-metropolitan areas [37]. Future pipeline programs including targeted outreach and mentorship programs may encourage recruitment from underrepresented groups to enter the OGP workforce [38]. Second, programmatic efforts are needed to address professional burnout and promote retention [28]. Increasingly, younger physicians value work-life balance and are interested in flexible scheduling options including locum tenens contracts and other job-sharing models to reduce burnout [31]. Additionally, support and wellness programs for OGPs may help mitigate stress and burnout [39]. Heath policy efforts are needed to advocate for medical malpractice reform including legal protections for obstetric care providers especially in high-risk and low resource settings [29]. Further efforts to promote late career transitions for OGPs including normalizing a transition to non-surgical care may serve to reduce OGP workforce attrition [30]. Third, there remains a need to create collaborative practice agreements between OGPs and advanced practice providers (APPs) to promote team-based care delivery models especially in underserved areas [40]. Further training and integration of nurse practitioners, certified nurse midwives, and physician assistants can enhance reproductive and gynecologic care delivery in areas with OGP workforce deficiency [41]. Fourth, expansion of telemedicine services can help enhance prenatal care delivery, contraceptive counseling, and follow-up gynecologic care in remote and underserved areas [42]. Additionally, technological advancements in artificial intelligence may have the capacity to improve the efficiency of the future OGP workforce [43]. However, the expansion of telemedicine services will require cross-state medical licensing reform and policies to ensure consistent reimbursement from government insurance plans [42]. Fifth, continued physician advocacy efforts by professional societies like ACOG and AGOS are needed to protect the specialty from declining insurance reimbursements from Medicare and Medicaid for obstetric and gynecologic services [44]. Further erosion of insurance payments from CMS despite rising practice expenses, has the potential to destroy access to obstetric and gynecologic care for an increasing volume of disadvantaged patient populations [45].

**Table 2:** Summary of potential strategies to improve obstetrics and gynecology physician workforce adequacy

Potential strategy	Description	Potential impact	Requirements for implementation
Increase in training capacity	New residency training positions and programs in obstetrics and gynecology	Increased annual supply of OGPs in areas of strategic interest	Coordination with educational regulatory bodies (e.g., ACGME) and identification of hospitals for residency expansion
Wellness initiatives	Programmatic efforts to reduce burnout and attrition in obstetrics and gynecology	Increased retention in the OGP workforce	Physician resources to support OGPs (e.g., mental health services)
Muti-disciplinary care delivery	Further integration of advanced practice providers in obstetrics and gynecology	Increased access to routine obstetric and gynecologic care in areas of identified deficiency	Training resources and requirements for nurse practitioners, certified nurse midwives, and physician assistants
Expansion of telemedicine services	Technology enabled delivery of routine obstetric and gynecologic care	Virtual-care delivery of OGP services across underserved areas	Cross-state legislation to recognize OGP credentialing for telemedicine services
Physician advocacy efforts	Preservation of the attractiveness of obstetrics and gynecology as a specialty	Increased recruitment and retention of future OGPs	Physician advocacy efforts to promote the specialty via insurance reimbursement and medical malpractice reform

OGP, obstetrics and gynecology physician; ACGME, Accreditation Council for Graduate Medical Education

This study had several strengths and limitations. This was a cross-sectional analysis of the US OGP workforce using data and assumptions inherent in the HWSM. Results from this study summarize the latest projections of the US federal government regarding anticipated shortages of OGPs across the US, which may have important policy implications. All states were accounted for in OGP workforce projections to 2037, yet these projections may be susceptible to future evolutions in obstetric and gynecologic care delivery, which are influenced by technological advancements, economic factors, and policy changes. Increasingly, obstetrics and gynecology are practiced in a sub-specialized manner [46], and specific OGP workforce trends in maternal fetal medicine, gynecologic oncology, and reproductive endocrinology and infertility were not available for analysis. Additionally, all insights were limited to the state level, and future studies are needed to understand OGP workforce disparities at the county and zip code levels. Lastly, clinical outcomes were not assessed in this study. Future studies are needed to correlate disparities in OGP workforce adequacy with maternal and neonatal outcomes [47], for example. Key stakeholders in the specialty, including ACOG and AGOS, should routinely monitor workforce dynamics through more granular workforce assessments of OGPs throughout the US.

In summary, the supply of OGPs is projected to decline, while the demand for their services is expected to increase. These diverging forces are expected to decrease the adequacy of the OGP workforce, especially in non-metropolitan areas, the West, and certain identified states such as Utah and Idaho. Collectively, the results from this study highlight an emerging deficiency in the number of OGPs in the US, which may have several policy implications moving forward. Future work is needed to develop strategies that enhance OGP workforce adequacy in geographic areas with identified deficiencies.

## Data Availability

The datasets used and/or analysed during the current study are available from the corresponding author on reasonable request.
